# Metabolite reanalysis revealed potential biomarkers for COVID-19: a potential link with immune response

**DOI:** 10.2217/fmb-2021-0047

**Published:** 2021-05-11

**Authors:** Xin Chen, Mingli Gu, Tengda Li, Yi Sun

**Affiliations:** ^1^Department of Laboratory Medicine, Shanghai General Hospital, Shanghai Jiao Tong University School of Medicine, Shanghai, 200080, China; ^2^Department of Laboratory Diagnosis, Changhai Hospital, Navy Military Medical University, Shanghai, 200433, China; ^3^State Key Laboratory of Experimental Hematology, National Clinical Research Center for Blood Diseases, Institute of Hematology & Blood Diseases Hospital, Chinese Academy of Medical Sciences & Peking Union Medical College, Tianjin, 300020, China; ^4^Princeton High School, Princeton, NJ 08540, USA

**Keywords:** biomarkers, COVID-19, immune response, metabolomic profiling

## Abstract

**Aim:** To understand the pathological progress of COVID-19 and to explore the potential biomarkers. **Background:** The COVID-19 pandemic is ongoing. There is metabolomics research about COVID-19 indicating the rich information of metabolomics is worthy of further data mining. **Methods:** We applied bioinformatics technology to reanalyze the published metabolomics data of COVID-19. **Results:** Benzoate, β-alanine and 4-chlorobenzoic acid were first reported to be used as potential biomarkers to distinguish COVID-19 patients from healthy individuals; taurochenodeoxycholic acid 3-sulfate, glucuronate and N,N,N-trimethyl-alanylproline betaine TMAP are the top classifiers in the receiver operating characteristic curve of COVID-severe and COVID-nonsevere patients. **Conclusion:** These unique metabolites suggest an underlying immunoregulatory treatment strategy for COVID-19.

The world is in the midst of the COVID-19 pandemic caused by the new SARS-CoV-2. According to Johns Hopkins Coronavirus Resource Center, more than 19 million people worldwide have been infected. It was estimated that approximately 60% of cases were nonsevere, but the severe cases may lead to critical results, such as death and serious sequela [1]. Approximately 5% of COVID-19 patients and 20% of hospitalized patients have severe symptoms and require intensive care. Among patients hospitalized in the intensive care unit (ICU), the fatality rate is as high as 40% [2]. The increasing number of infected cases has debilitated the public health system, affecting people's daily lives and global economic activity in an unprecedented fashion. Although emergency vaccines have been urgently approved for use, more clinical trials are still needed regarding their safety and effectiveness [2]. Therefore, on the basis of previous clinical diagnosis and treatment experience, strengthening the basic and clinical research of COVID-19 will help to achieve effective clinical intervention, curb transmission and reduce mortality. There is an urgent need to understand the pathological mechanism of COVID-19 and explore effective treatment methods.

Mass spectrometry brought the dawn of ‘omics’ sciences and is widely used in the analysis of complex mixtures, such as proteomics and metabolomics [3]. At the end of the omics cascade, metabolome acts as the interface between the genome and the environment and therefore is a useful phenotype indicator. In addition, the greater temporal sensitivity compared with other omics makes it an attractive approach to probing transient phenotypic changes caused by sudden incidents. Unlike immunoassays, mass spectrometry does not require antibodies, and can analyze a wide range of substances with high reproducibility and low limit of quantitation [4,5]. The features of metabolites, lipoproteins, proteomic and amino acids in COVID-19 patients were determined by nuclear magnetic resonance spectroscopy (NMR) and liquid chromatography–mass spectrometry (LC-MS) [5–9]. The studies of NMR by Lodge *et al*. [5], revealed that plasma supramolecular phospholipid composite signals (SPC total) and SPC total/GlycA are proposed as sensitive molecular markers for SARS-CoV-2 positivity and in functional assessment of the disease recovery process in patients with long-term symptoms. They analyzed 1H NMR spectroscopy data in human plasma and modeled them together with a variety of plasma cytokines and chemokines. The results [6] showed a unique pattern for SARS-CoV-2-infected cells with multiple levels. The immune response interacts with the plasma lipoprotein group to give a strong and unique immune metabolic phenotype of the disease. Meoni *et al*. [7] analyzed the metabolomics and lipidomics of COVID-19 patients and showed that COVID-19 patients had characteristic NMR-based metabolomic and lipidomic characteristics. There was an exploratory study [8] on 162 metabolites in the plasma of ICU patients (both COVID-19+ and COVID-19-) and the data was analyzed using advanced machine learning. A unique COVID-19 plasma metabolome was discovered, which is mainly determined by the changes in kynurenine, arginine, sarcosine and LysoPCs. Moreover, creatinine alone or the creatinine/arginine ratio can predict ICU mortality with 100% accuracy, suggesting that metabolites (kynurenine, arginine and creatinine) can be regarded as potential biomarkers and prognostic markers for diagnosis of COVID-19. Guo *et al*. [9] compared metabolomic and proteomic profiles of serum samples obtained from COVID-19 patients with that of healthy volunteers and symptomatic patients diagnosed with non-COVID-19 disease control. From their results, they discovered protein and metabolite dysregulation in severe COVID-19 patient sera, which may contribute to macrophage modulation. The application of bioinformatics to clinical medicine can improve the accuracy of diagnosis and treatments. We applied bioinformatics technology to reanalyze the previously published metabolomics data of COVID-19 patients. Previous studies [9] on the characteristics of plasma metabolism in patients with COVID-19 have found that the proteins and metabolites in the serum of patients with severe COVID-19 are imbalanced, which may contribute to the regulation of macrophages. This integration of proteomics and metabolomics methods provides a view of the underlying pathology of disease progression, but the rich information in metabolomics may be overlooked. Therefore, it is worthy of more detailed research.

Herein, we conducted further statistical analysis on the data from the previously reported metabolomics studies. Our analysis suggests that COVID-19 infection affected the cell signal, nucleic acid metabolism and amino acid metabolism networks in the COVID-19 patients. Metabolites, including benzoic acid, phosphate and inosine, were first reported to significantly increase in sera from COVID-19 patients, promoting immune response and inflammation development, contributing to damage of multiple tissues and organs such as lung, liver and kidney. The metabolomics profiles of COVID-19 patients were also distinct from the disease control group, suggesting a clue to targeted treatment strategy for nonsevere and severe patients.

## Methods

### Data source

The data used in the study was retrieved from the CoronaMassKB database (dataset ID MSV000085507) which consisted of 25 samples from nonsevere patients diagnosed with COVID-19, 21 samples from severe patients diagnosed with COVID-19, 25 samples from healthy individuals and 25 samples from non-COVID-disease control patients. The detailed patient descriptions including the sampling date and the metabolite data for each patient can be found in the original paper [9] and are shown in Supplementary Tables 1–3. According to the source information, 65 COVID-19 patients were classified into four subgroups based on the Chinese Government Diagnosis and Treatment Guideline (Trial 5th version) [10]. Mild: mild symptoms without pneumonia; typical: fever or respiratory tract symptoms with pneumonia; severe: fulfill any of the three criteria: respiratory distress, respiratory rate R 30 times/min; means oxygen saturation 93% in resting state; arterial blood oxygen partial pressure/oxygen concentration 300 mmHg (1 mmHg = 0.133 kPa); critical: fulfilling any of the following three criteria: respiratory failure and requirement for mechanical ventilation, shock incidence or admission to ICU with other organ failure [9]. The mild and typical subgroups made up the nonsevere subgroup. The disease control group in the study consisted of 25 non-COVID-19 patients with similar clinical characteristics including fever and/or cough as COVID-19 patients, but tested negative for COVID-19 [9]. Causal analysis of the infection showed that four patients were infected by herpes simplex virus, one patient infected by varicella-zoster virus, one by respiratory syncytial virus, one by *Klebsiella pneumoniae* and *Acinetobacter baumannii* and one by *Enterococcus faecium* [9]. Some patients had other diseases, including cancer, cerebral hemorrhage or lymphoma [9]. No infection was detected in the other patients according to respiratory tract virus antigen test [9]. The healthy group included serum samples from 28 healthy individuals [9].

### Statistical analysis

We performed a two-sample *t*-test on COVID-19 patients' group and the healthy group to understand the pathology of the disease, followed by another two-sample *t*-test to distinguish the COVID-19 patients' group from the disease control group; finally, we examined the difference between the severe and nonsevere subgroups to predict disease progression. Statistical analysis was performed on MetaboAnalyst (https://www.metaboanalyst.ca/) a metabolomics analysis platform that has integrated R scripts [11–23]. Missing values were estimated using k-nearest neighbors. Metabolites with missing values in 50% or more samples were excluded. Subsequently, the data were normalized by sum of the total ion intensity, log transformed and scaled using Pareto scaling to facilitate the downstream hypothesis testing. In the subsequent two-sample *t*-test of each group, metabolites with a concentration change greater than twofold and a false discovery rate-adjusted p-value <0.05 were considered significantly altered. For hierarchical clustering analysis, the distance measure was Euclidean, and the clustering algorithm was ward. Classification using random forest and subsequent characteristic (ROC) analysis was carried out using seven predictors and up to 500 trees. To analyze the disease-related metabolic pathways and cell signaling networks, pathway analysis was performed using Ingenuity Pathway Analysis (IPA), which is a cloud computing-based bioinformatics software utilising integrated metabolomics analysis for the over-represented metabolic pathways.

## Results

### Metabolomics profiles of COVID-19 patients were significantly different from those of the healthy group

**Figure 1.**
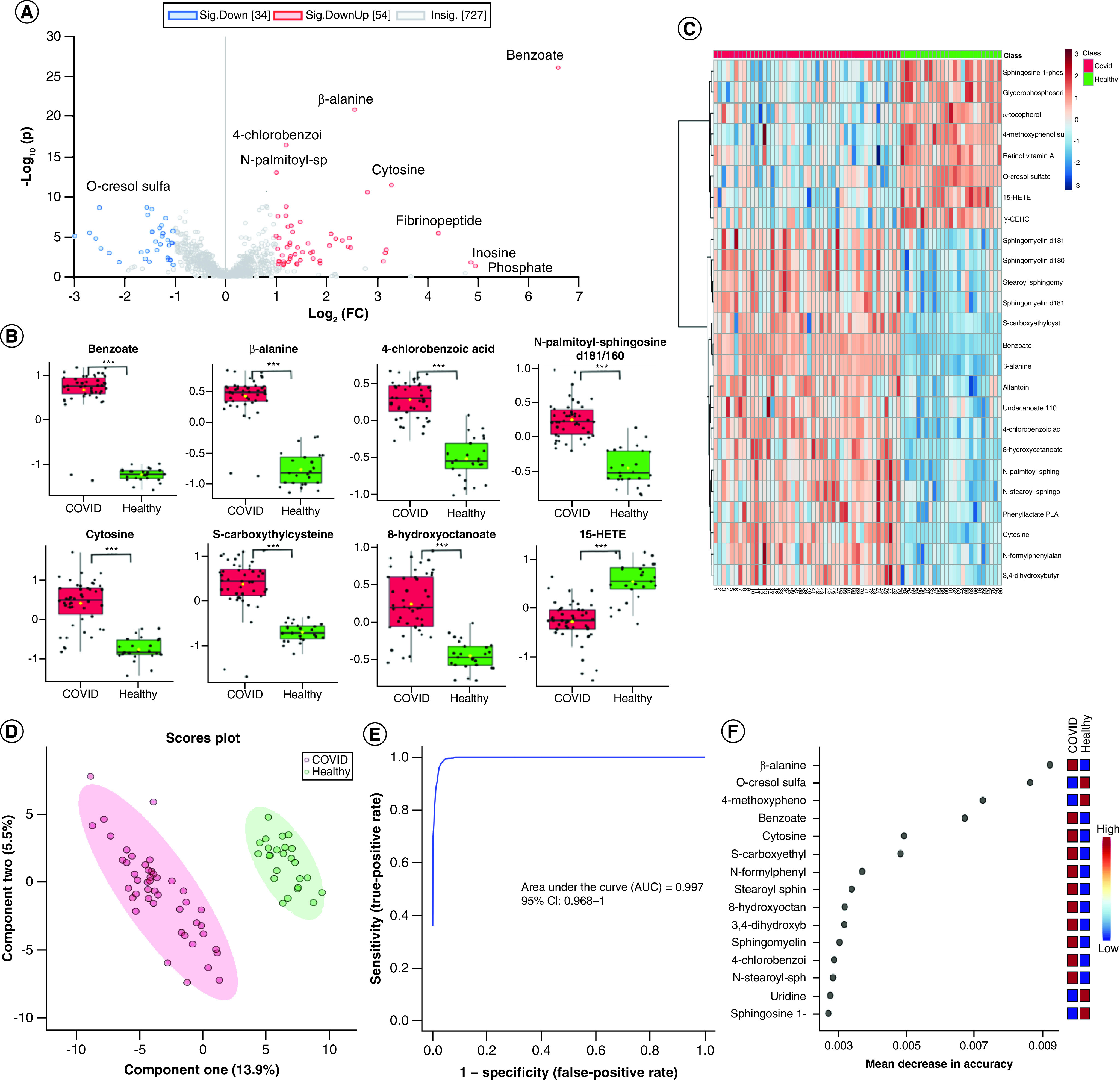
Comparative analysis of the plasma metabolome could differentiate significantly COVID-19 patients and healthy group. **(A)** Volcano plot significantly separated COVID-19 patients and the healthy group, purple dots are the compounds that are both significant in adjusted p-value and fold change and black dots are the compounds that are not significant. **(B)** The expression level change (Z-scored original value) of eight selected metabolites with significant difference. Asterisks indicate statistical significance based on unpaired two-sided Welch's *t*-test. Red boxes are COVID-19 patients, green boxes are healthy individuals and the y axis is the normalized fold change. **(C)** Heat map of selected average plasma metabolite expression levels. **(D)** COVID-19 patients and healthy group were separated by components one and two in the PLS-DA machine learning analysis. **(E)** Receiver operating characteristic using five metabolites as classifiers. **(F)** Fifteen metabolites prioritized by random forest analysis ranked by the mean decrease in accuracy. *p < 0.05; **p < 0.01; ***p < 0.001.

We first conducted the two-sample *t*-tests of metabolites between COVID-19 patients and healthy individuals. The analysis revealed that COVID-19 patients' plasma metabolomic profiles were distinctly different from those of healthy individuals (Figure 1A). Using the cutoff of adjusted p < 0.05 and fold change >2 (the same thereafter), 88 metabolites were identified as being significantly changed in COVID-19 patients. Twenty of the 88 significantly altered metabolites were mapped into metabolic pathways to identify overrepresented pathways, which were discussed in detail later. The concentration changes of eight altered metabolites with the most significantly adjusted p and fold change is shown in Figure 1B. A heat map using the top 25 differentially produced metabolites demonstrated a clear difference between COVID-19 patients and healthy individuals (Figure 1C). The partial least squares discriminant analysis (PLS-DA) successfully separated the COVID patients from healthy individuals (Figure 1D). ROC analysis based on random forest yielded an area under the curve (AUC) of 0.997 when the five most significant metabolites are used as classifiers (95% CI: 0.968–1) (Figure 1E), indicating the practicality of using metabolite biomarkers to differentiate COVID from healthy individuals. On the basis of the mean decrease in the accuracy of the random forest classification, the top three biomarkers that can be used to differentiate COVID-19 patients from healthy individuals were β-alanine (Q = 1.26 × 10^-21^), o-cresol sulfate (Q = 2.38 × 10^-9^), 4-methoxyphenol sulfate (q = 3.77 × 10^-9^) (Figure 1F).

**Figure 2.**
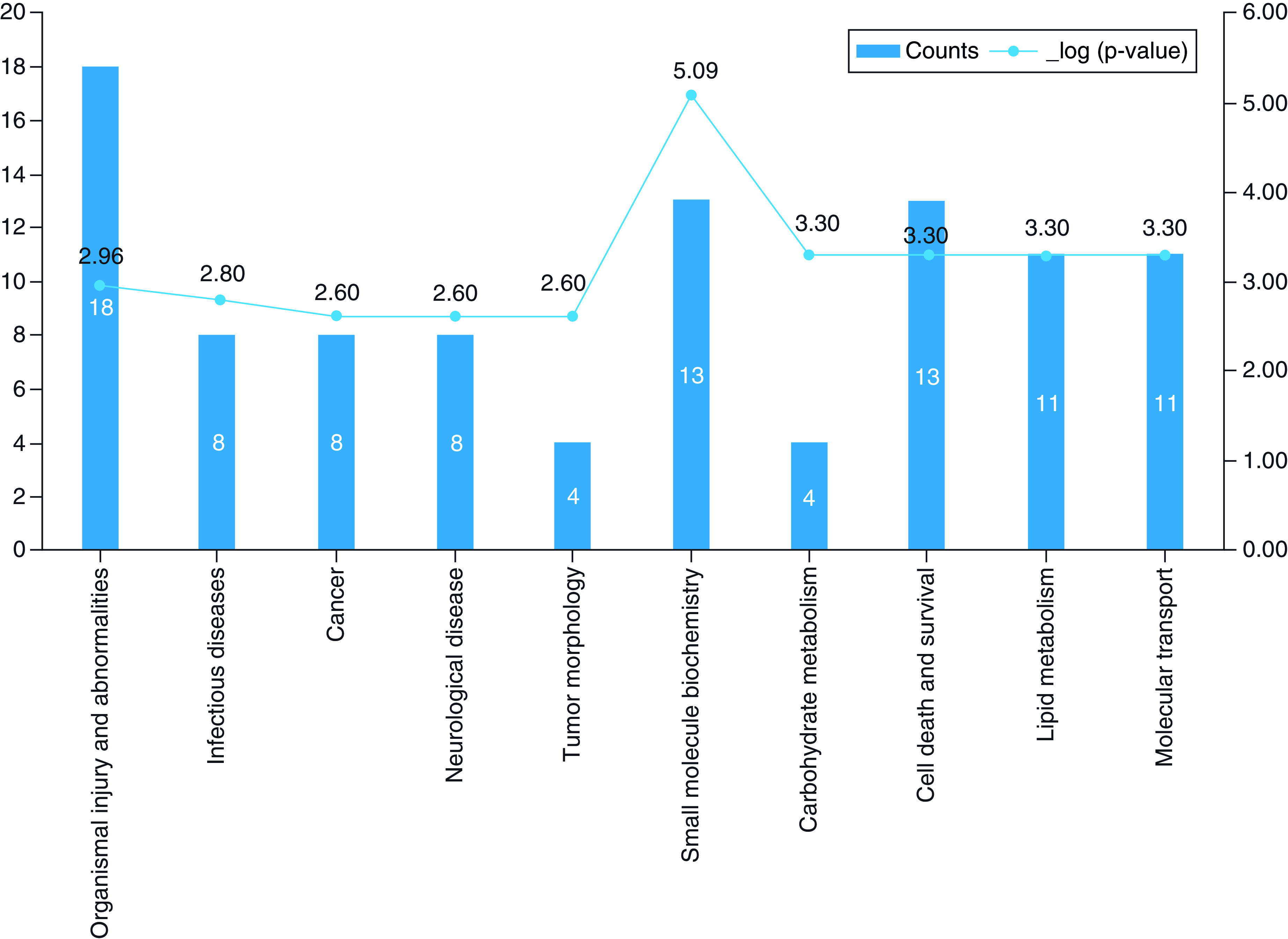
Network of prioritized metabolites in the COVID-19 patients. Red and green nodes indicate upregulated and downregulated molecules, respectively. White nodes represent molecules not detected in our dataset.

Ingenuity pathway analysis indicated alteration of purine ribonucleotides degradation (p = 4.19 × 10^-4^), purine nucleotides degradation II (aerobic) (p = 1.24 × 10^-3^), salvage pathways of pyrimidine (p = 1.24 × 10^-3^). Furthermore, network analysis of prioritized metabolites in the COVID-19 patients (Figure 2) suggested that the networks of cell-to-cell signaling and interaction (score = 30) and nucleic acid metabolism and amino acid metabolism (score = 24) played a very active regulatory role in COVID-19.

### Metabolomics profiles of COVID-19 patients were distinct from those of the disease control group

**Figure 3.**
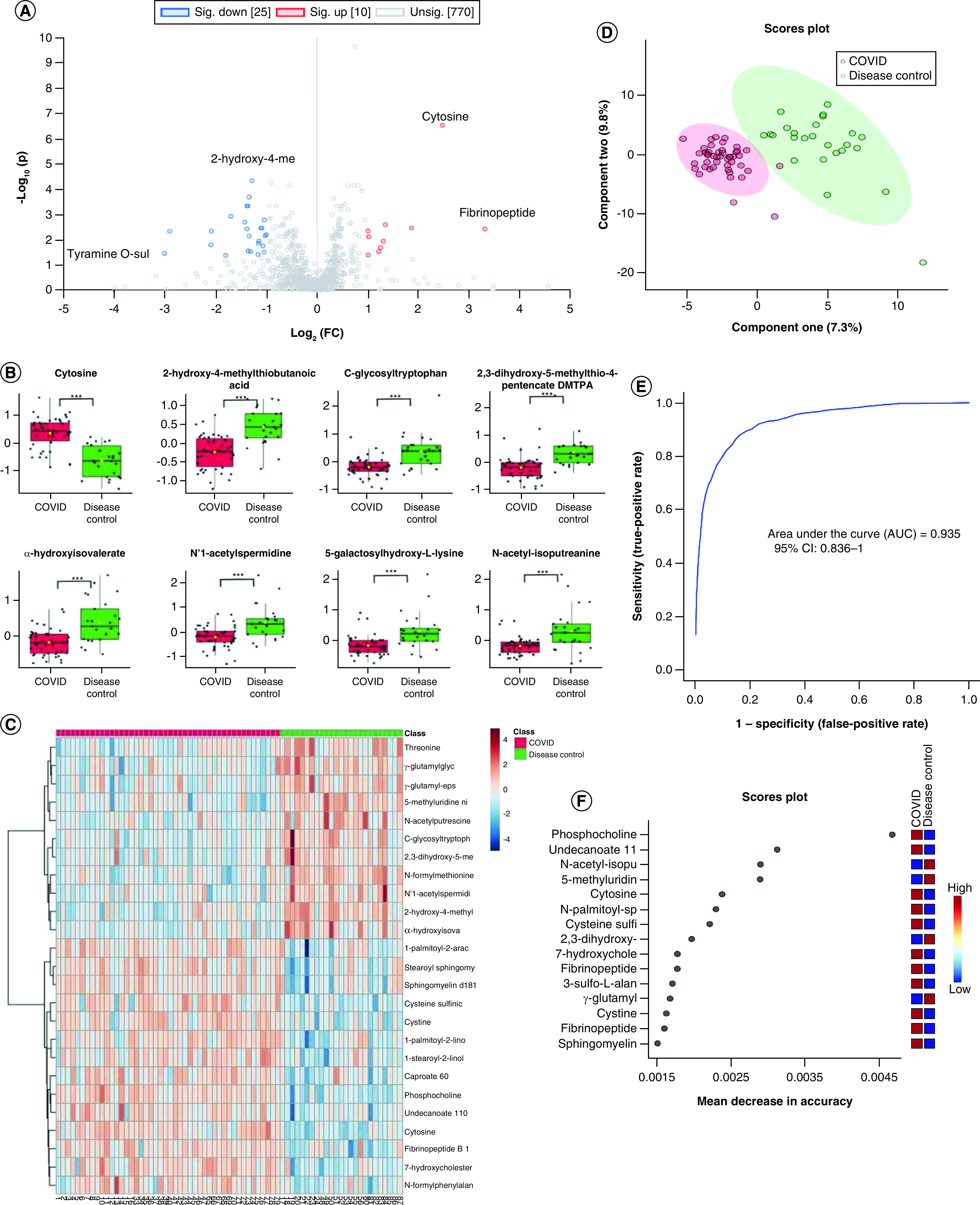
Two-sample t-test analysis of the plasma metabolome of COVID-19 patients and non-COVID disease control patients. **(A)** Volcano plot significantly separated COVID-19 patients and non-COVID patients; purple dots indicate both significant fold change and adjusted p-values. **(B)** The expression level change (Z-scored original value) of eight selected metabolites with significant fold change and adjusted p-values. Asterisks indicate statistical significance based on unpaired two-sided Welch's *t*-test. Green boxes are disease control sample, and red boxes are COVID-19 samples. **(C)** Heat map of selected average plasma metabolite expression levels. **(D)** COVID-19 patients and non-COVID groups were separated by component 1 and 2 in the partial least squares discriminant analysis machine learning analysis. **(E)** Receiver operating characteristic with five metabolites. **(F)** Fifteen metabolites prioritized by random forest analysis ranked by the mean decrease in accuracy. *p < 0.05; **p < 0.01; ***p < 0.001.

**Figure 4.**
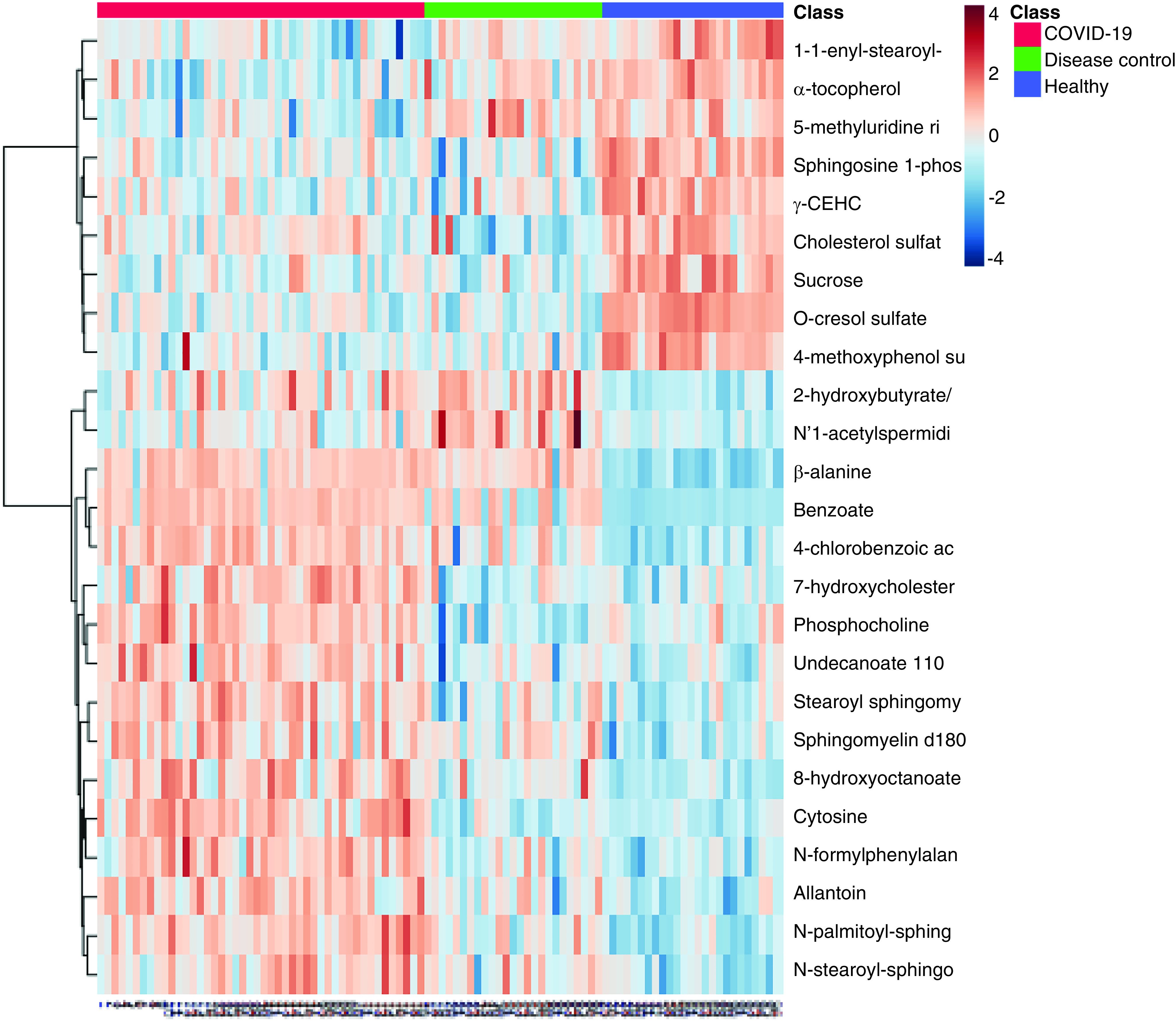
Heat map of the plasma metabolome of COVID-19 patients, non-COVID disease control patients and healthy participants.

Two-sample *t*-test between COVID-19 patients and non-COVID disease control samples was carried out to identify biomarkers that could be used to differentiate the two pathological conditions. The result suggested that COVID-19 patients' plasma metabolomics profiles were different from those of disease controls but to a lesser extent than the comparison between COVID-19 and healthy individuals (Figure 3A). On the basis of the criteria described earlier, 35 metabolites were identified as significantly changed, and the differential production of eight metabolites with most significant fold change and adjusted p-value is shown in Figure 3B. Hierarchical clustering using top 25 significant metabolites revealed that there was moderate distinction between the metabolomics profiles of the two groups (Figure 3C). Meanwhile, based on PLS-DA analysis, the COVID-patients and disease control samples were largely distinguishable (Figure 3D), although less so compared to COVID versus healthy analysis (Figure 4). Finally, ROC curve based on a random forest using five metabolites as classifiers resulted in an AUC of 0.935 (95% CI: 0.836–1) (Figure 3E). In random forest analysis, we listed top 15 metabolites with marked mean decreases in accuracy. On the basis of the mean decrease in accuracy of the random forest classification, the top three biomarkers with significant adjusted p-value that could be used to differentiate COVID-19 patients from disease control patients were cysteine sulfinic acid (Q = 7.118 × 10^-5^), phosphocholine (Q = 2.253 × 10^-10^), 3-sulfo-L-alanine (Q = 0.00164) (Figure 3F).

### Selected metabolites profiles could differentiate severe group and non-severe group COVID-19 patients

**Figure 5.**
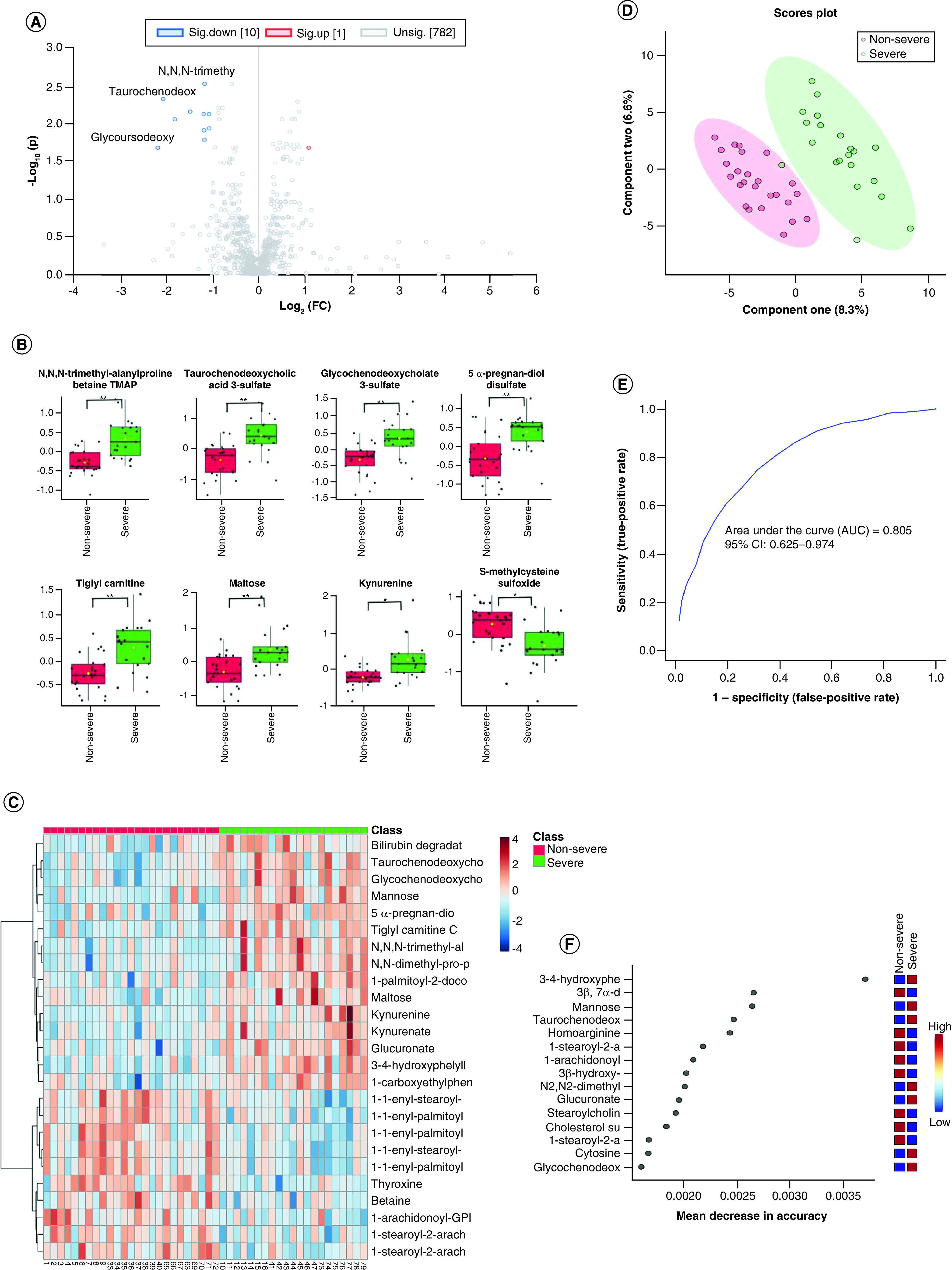
Comparative analysis of the plasma metabolome could differentiate the severe and nonsevere COVID-19 patient groups. **(A)** Volcano plot of the nonsevere and severe groups; purple dots are significant in fold change and adjusted p-value. **(B)** The expression level change (Z-scored original value) of eight selected metabolites with significant adjusted p-values and fold change; green boxes are the severe sample, and red boxes are the nonsevere sample. Asterisks indicate statistical significance based on unpaired two-sided Welch's *t*-test. **(C)** Heat map of selected average plasma metabolite expression levels. **(D)** Severe and nonsevere groups were separated with component one and two in partial least squares discriminant analysis machine-learning analysis. **(E)** Receiver operating characteristic with ten metabolites. **(F)** Fifteen metabolites prioritized by random forest analysis ranked by the mean decrease in accuracy. *p < 0.05; **p < 0.01; ***p < 0.001.

To determine the practicality of using biomarker to assess disease progression, we examined the metabolomes from severe and nonsevere patients in the volcano plot (Figure 5A). On the basis of the same criteria described earlier, 11 metabolites were noted as significantly altered, of which nine are shown in Figure 5B. PLS-DA analysis showed that the severe and nonsevere cases could be separated by differentially regulated metabolomes (Figure 5D). The differences between the metabolomics profiles of severe and nonsevere COVID-19 patients were revealed in the heat map (Figure 5C). The differential metabolomics profile was also analyzed by ROC analysis (0.805; 95% CI: 0.625–0.974) using ten classifiers (Figure 5E), and a number of differential metabolites with significantly adjusted p-values were identified, such as taurochenodeoxycholic acid 3-sulfate (Q = 0.00479), 5α-pregnan-diol disulfate (Q = 0.00762) and N,N,N-trimethyl-alanylproline betaine TMAP (Q = 0.00302) (Figure 5F).

## Discussion

We first reported that the metabolites benzoate, β-alanine and 4-chlorobenzoic acid to be used as potential biomarkers to distinguish COVID-19 patients from healthy individuals with an AUC of 0.997 (95% CI: 0.988–1). Further analysis of severe and nonsevere COVID resulted in an ROC curve with an AUC of 0.805 (95% CI: 0.625–0.974), and the top classifiers were taurochenodeoxycholic acid 3-sulfate, glucuronate and N,N,N-trimethyl-alanylproline betaine TMAP. In the comparison of the ROC analysis of healthy and COVID-19 groups and non-COVID-19 and COVID-19 patients mentioned earlier, differentiating severe from nonsevere COVID-19 patients was more challenging but still generated a fair result [24]. In general, the ROC analysis established in our research can distinguish severe from nonsevere patients, which can provide an important basis for personalized and precise treatment. The immediate analysis of these metabolites can be developed rapidly, and patient stratification is essential for future COVID-19 treatment drug trials. The results of these studies on the metabolome need to be verified in a larger COVID-19 population before they are useful.

Among the differentially produced metabolites found in patients carrying COVID-19, benzoic acid, phosphate, inosine and sucrose have been suggested to promote immune response and inflammation development [25–27]. (R)-3-hydroxybutyric acid was correlated to phagocytosis cell damage [28]. It was also reported that N-formyl phenyl alanine chemotaxis can regulate the aggregation of human neutrophils [29]. The aforementioned metabolites were all inflammation-promoting factors and were all significantly increased in samples from COVID-19 patients, indicating an important role of inflammation in COVID-19 disease progression. There was a significant increase in cytosine and ribose, indicated by the overrepresentation of purine ribonucleotides degradation. The upregulations of purine nucleotides degradation II (aerobic) and other nucleic acid metabolome pathways were indicators of inflammation [30]. On the other hand, β-alanine, tauroursodeoxycholic and taurocholic acid were also elevated in COVID-19 patients, which have been suggested to be involved in the negative feedback of the inflammation that plays a certain role in immune regulation [31–33].

More negative feedback modulators were decreased, such as lenticin, which contained a protective mechanism during the damage to maintain the stability of the internal environment [34], 15(S)-HETE, which was the negative feedback regulator of immune response [35], 2,7,8-trimethyl-2-(β-carboxyethyl)-6-hydroxychroman, which was the peroxy free radical scavenger that maintains stability [36]. Their disorder served as a potential indicator that some negative feedback modulators were overconsumed in COVID-19 patients, which made the inflammatory response control ineffective.

The recent study [8] on critically ill COVID-19 patients showed that there are unique metabolomes in the plasma of COVID-19 ICU patients, including kynurenine, arginine, sarcosine and LysoPCs. They proposed a diet supplementation of tryptophan, arginine, sarcosine and LysoPCsas adjuvant therapy may contribute to COVID-19 outcome. Our study has similar results, such as increased kynurenine, suggesting that the immune response is overactivated. Activating COVID-19 causes strong T-cell activation, and IFN-γ rises, which in turn causes the degradation of tryptophan to increase, and kynurenine also increases. Targeting metabolism markedly modulates the proinflammatory cytokines release by peripheral blood mononuclear cells isolated from SARS-CoV-2-infected rhesus macaques *ex vivo* according to the most recent study on fatal cytokine-release syndrome in COVID-19 [37]. The results suggest that patients' immune regulatory mechanism may be a potential therapeutic target for COVID-19. Clinical treatment also showed that suppressing inflammation can help alleviate disease symptoms [38]. These unique metabolites are accurate diagnostic/prognostic biomarkers for future research, representing a variety of metabolites that affect immune function and can be used for stratified evaluation of patients in clinical treatment.

## Conclusion

Together, the analysis results revealed that COVID-19 infection affected patients' cell signal, nucleic acid metabolism and amino acid metabolism networks. Our research on the COVID-19 metabolome pathways contributed to the understanding of the role of immune regulatory pathways during viral infection, which will also serve as an important therapeutic target for more effective treatment of COVID-19.

## Future perspective

Although vaccines are bringing hope to the fight against the COVID-19 virus, we have experienced a third wave of the pandemic involving variants of COVID-19. Therefore, exploring biomarkers related to COVID-19 disease has important long-term significance for appropriate personalized treatment and prevention of COVID-19. Studies have shown that with regard to the pathogenesis of COVID-19, in addition to direct virus invasion and damage to target tissues, the increase in inflammatory biomarkers such as C-reactive protein reflects the development of the disease. Moreover, the metabolomics research on COVID-19 suggests that some characteristic metabolites can be used as biomarkers related to COVID-19, such as SPC total and SPC total/GlycA, kynurenine, arginine and creatinine, forming a unique immune metabolic phenotype. However, due to the large number of molecules involved, in further research, mathematical models could be established by deep learning, taking into account various factors related to the disease to guide more accurate application of antiinflammatory and immunostimulatory activities, reducing the severity of the disease and preventing multiple organ failure and death. In addition, it also has guiding significance for diagnosis and treatment of other viral infections in the future.

**Summary points**

- Two-sample *t*-tests on COVID-19 patients and healthy participants to understand the pathology of the disease were conducted, followed by two-sample t-tests to distinguish COVID-19 patients from a disease control group and differentiate severe and nonsevere subgroup of COVID-19 patients.
- Pathway analysis was performed using Ingenuity Pathway Analysis (IPA), a cloud computing-based bioinformatics software with integrated metabolomics analysis for the overrepresented metabolic pathways.
- The metabolomics profiles of COVID-19 patients were significantly different from those of the healthy group. Receiver operating characteristic (ROC) analysis based on a random forest plot yielded an area under the curve (AUC) of 0.997 when the five most significant metabolites were used as classifiers (95% CI: 0.968–1), indicating the practicality of using metabolite biomarkers to differentiate COVID patients from healthy individuals. The top three biomarkers that can be used to differentiate COVID-19 patients from healthy individuals were β-alanine, o-cresol sulfate and 4-methoxyphenol sulfate.
- IPA indicated alteration of purine ribonucleotides degradation, purine nucleotides degradation II (aerobic) and salvage pathways of pyrimidine. Network analysis suggested that the networks of cell-to-cell signaling and interaction, nucleic acid metabolism and amino acid metabolism played active regulatory roles in COVID-19.
- The metabolomic profiles of COVID-19 patients were distinctive from disease control group. An ROC curve based on a random forest plot using five metabolites as classifiers resulted in an AUC of 0.935 (95% CI: 0.836–1). The top three biomarkers with significant adjusted p-values that can be used to differentiate COVID-19 patients from disease control patients were cysteine sulfinic acid, phosphocholine and 3-sulfo-L-alanine.
- The selected metabolites profiles could differentiate severe and nonsevere subgroups of COVID-19 patients. Partial least squares discriminant analysis showed that the severe and nonsevere cases could be separated by differentially regulated metabolomes. The differential metabolomics profile was also analyzed by ROC analysis (0.805, 95% CI 0.625–0.974) using ten classifiers and a number of differential metabolites with significantly adjusted p-values were identified, such as taurochenodeoxycholic acid 3-sulfate, 5α-pregnan-diol disulfate and N,N,N-trimethyl-alanylproline betaine TMAP.
- β-alanine, o-cresol sulfate, 4-methoxyphenol sulfate, taurochenodeoxycholic acid 3-sulfate, 5α-pregnan-diol disulfate and N,N,N-trimethyl-alanylproline betaine TMAP could be biomarkers in COVID-19, which contributes to understanding the role of immune regulatory pathways during infection and could serve as an important therapeutic target for treatment of COVID-19.
