# Supplementary figures and images for: Metabolite reanalysis revealed potential biomarkers for COVID-19: a potential link with immune response
Source: Future Microbiol. 2021 May 11:10.2217/fmb-2021-0047. doi: 10.2217/fmb-2021-0047 (PMC8112156; doi:10.2217/fmb-2021-0047)

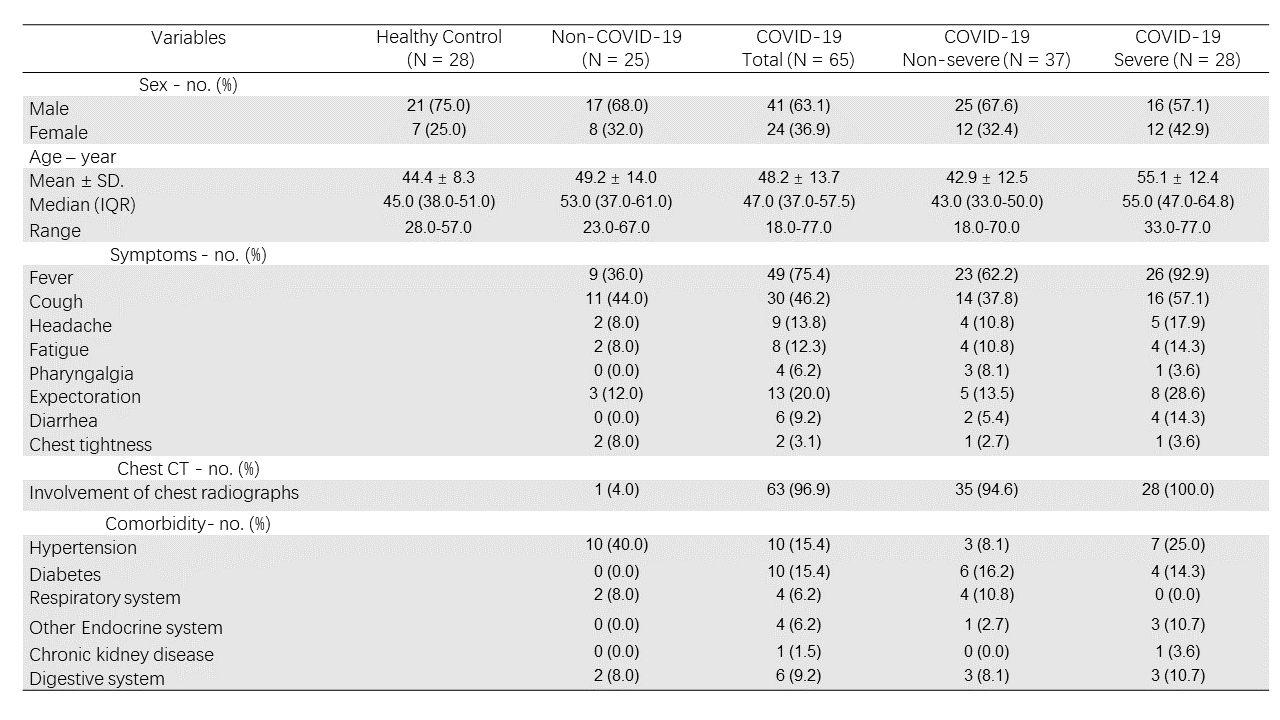

Supplement: Supplementary file 1 [file Table_S1.png]
